# Adoptive Transfer of Regulatory Immune Cells in Organ Transplantation

**DOI:** 10.3389/fimmu.2021.631365

**Published:** 2021-03-02

**Authors:** Nathaniel Oberholtzer, Carl Atkinson, Satish N. Nadig

**Affiliations:** Department of Surgery, Medical University of South Carolina, Charleston, SC, United States

**Keywords:** transplantation, solid organ transplant, regulatory T cells, myeloid derived suppressive cells, chimeric antigen receptor, immunoengineering, graft rejection, IL-10-producing B cells Bregs

## Abstract

Chronic graft rejection remains a significant barrier to solid organ transplantation as a treatment for end-organ failure. Patients receiving organ transplants typically require systemic immunosuppression in the form of pharmacological immunosuppressants for the duration of their lives, leaving these patients vulnerable to opportunistic infections, malignancies, and other use-restricting side-effects. In recent years, a substantial amount of research has focused on the use of cell-based therapies for the induction of graft tolerance. Inducing or adoptively transferring regulatory cell types, including regulatory T cells, myeloid-derived suppressor cells, and IL-10 secreting B cells, has the potential to produce graft-specific tolerance in transplant recipients. Significant progress has been made in the optimization of these cell-based therapeutic strategies as our understanding of their underlying mechanisms increases and new immunoengineering technologies become more widely available. Still, many questions remain to be answered regarding optimal cell types to use, appropriate dosage and timing, and adjuvant therapies. In this review, we summarize what is known about the cellular mechanisms that underly the current cell-based therapies being developed for the prevention of allograft rejection, the different strategies being explored to optimize these therapies, and all of the completed and ongoing clinical trials involving these therapies.

## Introduction

At present, solid organ transplantation remains the only curative treatment for patients with end-stage organ disease. Organ transplantation has evolved over the past 60 years to become the predominant treatment option for end-organ failure, as advancements in immunosuppressive therapies have led to significantly reduced rates of acute organ rejection with improvement in 1-year graft survival (1). However, long-term survival of grafts and the prevention of chronic rejection has remained a significant hurdle in the success of solid organ transplant therapy. While the long-term survival rate of grafts has not seen significant improvement, the burden of lifelong immunosuppressive regimens contributes to the morbidity and mortality transplant recipients (1). The most commonly used maintenance immunosuppressive drugs used in solid organ transplant include steroids, calcineurin inhibitors (CNIs), antiproliferative agents (i.e. mycophenolate mofetil) and drugs that inhibit the mammalian target of rapamycin (mTOR). All of these drugs have drug-specific side-effects that can lead to nonadherence, as well as common use-restricting toxicities such as nephrotoxicity, increased cardiovascular risk, and systemic overimmunosuppression that can result in opportunistic infections as well as some malignancies (2). Given the significant side-effect burden of current immunosuppressive therapies and a persistent rate of chronic graft rejection, there is a need for minimization strategies that reduce (or eliminate) the amount of immunosuppressive drugs required for graft survival, with the ultimate goal being immunologic tolerance (i.e. stable graft tolerance in the absence of any systemic immunosuppression).

In solid organ transplantation, graft rejection occurs by two main pathways: the direct pathway and the indirect pathway. It is generally believed that the direct mechanism of T cell activation predominates early in graft rejection as there is an abundance of APCs present in the graft (i.e. donor passenger leukocytes), but that progressive depletion of the donor passenger leukocytes over time ultimately leads to a predominance of the indirect mechanism of T cell activation (3–7). Thus, it is the indirect pathway that ultimately persists leading to chronic graft rejection by priming effector T cells to induce cellular rejection, while also promoting a delayed-type hypersensitivity reaction that drives antibody-mediated rejection (AMR) and the inflammatory response of the innate immune system (5–10). Given this knowledge, it is logical to pursue adoptive cell-based therapies that have indirect allo-specificity to combat the progression of chronic allograft rejection and promote immune tolerance.

Much of the research involving the induction of graft tolerance has focused on cell-based therapies that use regulatory cell types belonging to both the innate and adaptive immune systems. Of particular interest have been regulatory T cells (Tregs), which were identified in a landmark study in 1995 showing a subpopulation of CD4^+^ T cells that expressed the IL-2 receptor (CD25) and were responsible for preventing the development of autoimmune disease (11). Other regulatory cell types have been identified, including myeloid-derived suppressor cells (MDSCs), immunosuppressive IL-10 secreting B cells (B10), tolerogenic dendritic cells (DCs), and natural killer cells (NKs). Each of these cell types act by distinct and sometimes synergistic methods, with varying degrees of promise for clinical utility in the setting of solid organ transplant. A number of studies have focused on either expanding these cell types *in vivo* in transplant recipients, while others have developed protocols for expanding regulatory cell types *ex vivo* and adoptively transferring them into transplant recipients (12–20). The bulk of the published research thus far has focused on Tregs, MDSCs, and B10 as the most promising candidates, and each of these potential therapeutic strategies are reviewed here.

## Regulatory Cell Types and How They Develop

### Regulatory T Cells

One of the most heavily investigated types of regulatory cells are a subset of CD4+ cells that primarily act to promote tolerance of both self and non–self-antigens, commonly known as Tregs. Naturally occurring Tregs are produced either in the thymus (central Tregs) or can be induced in the periphery (iTregs). While there is some heterogeneity in the markers expressed by specific subsets of Tregs, in both humans and mice they can generally be identified by co-expression of CD4 and CD25, as well as Foxp3 which serves as the “master regulator” for Treg development (21, 22). In their initial 1995 paper identifying the CD4^+^CD25^+^ Treg population, Sakaguchi et al. also showed that CD25 knockout mice exhibited heightened immune response to allogeneic skin transplantation, which could be normalized by reconstitution with CD4^+^CD25^+^ cells, collectively showing that CD4^+^CD25^+^ T cells (Tregs) are important for the maintenance of self-tolerance as well as tolerance to some non–self-antigens (11). The majority of human Tregs that maintain self-tolerance develop in the thymus, and their development is dependent on the strength and duration of T cell receptor (TCR) signaling, based on interaction with MHC-self peptides, as well as a combination of cytokines including IL-2, IL-15, and TGF-β (23–25). Of critical importance to Treg development in both humans and mice is selective demethylation of an element within the Foxp3 locus known as the Treg-specific demethylated region (TSDR) (26, 27). Studies in both humans and mice have demonstrated that epigenetic imprinting within this region is initiated during early stages of thymic Treg development, resulting in long-term stability of Foxp3 expression and commitment to the Treg lineage (24, 28). Fontenot et al. showed in a murine model that Foxp3 expression is required for both the development and suppressor function of Tregs, as Foxp3 knockout mice developed lethal autoimmune disease, and ectopic expression of Foxp3 was able to confer suppressor function to CD4^+^CD25^-^ T cells (29). Of note, Jeffrey Bluestone and colleagues showed in 2006 that CD127 (IL-7Rα) serves as an additional marker to differentiate highly suppressive human Tregs, as CD127 expression inversely correlates with suppressive capability (30). Nadig et al. built upon this finding by showing in 2010 that *ex vivo* expanded Tregs sorted based on low expression of CD127 (CD127^lo^) provide a more potent therapy compared to conventional Tregs in a humanized mouse system modeling transplant arteriosclerosis (12).

While Tregs that maintain self-tolerance primarily develop in the thymus, another population of CD4^+^Foxp3^-^ T cells in the periphery can be stimulated to become CD4^+^Foxp3^+^ Tregs primarily in response to non–self-antigens, termed induced Tregs (iTregs) (31). Using a murine model, Kretschmer et al. demonstrated that repeated, small antigen doses with suboptimal dendritic cell activation, along with the addition of TGF-β, resulted in increased conversion of these cell types (31). Multiple studies have investigated the signaling required for the induction of Tregs in the periphery, collectively showing that CD4^+^CD25^-^ cells coming from the thymus can be induced to become antigen-specific CD4^+^CD25^+^Foxp3^+^ iTregs by a combination of TCR signaling along with TGF-β and IL-2 signaling (32–34). In addition to promoting the differentiation of iTregs in the periphery, IL-2 also functions to inhibit the development of Th17 cells, thereby constraining the production of IL-17 and providing additional tolerogenic function (35). Using a murine model, Gottschalk et al. further elucidated the specific strength and duration of TCR stimulation that is required to induce Tregs in the periphery, and they found that low dose of a strong agonist in the setting of suboptimal co-stimulation provided the maximum stimulation for induction of Foxp3^+^ Tregs *in vivo* (33). This suggests that recognition of antigens by TCRs to which the organism has chronic exposure to leads to the differentiation of iTregs, resulting in tolerance.

There is a subpopulation CD4^+^CD25^-^ iTregs in humans known as T regulatory type 1 (Tr1) cells characterized by their ability to produce predominantly IL-10 and TGF-β and to transiently upregulate Foxp3 expression to induce tolerance (36). These cells are of special interest to the application of transplant therapy as they were first described in patients who developed tolerance after HLA-mismatched fetal liver hematopoietic stem cell transplantation and preliminary clinical trials have shown safety and efficacy of the use of these cells in human patients (36). While Tr1 cells are not as well-characterized as Tregs, it has been suggested that Tr1 cells can be differentiated in both humans and mice based on co-expression of CD49b and LAG-3 (37). The phenotypic markers that delineate Tregs and Tr1 cells in both mice and humans are summarized in **Table 1**.

### Myeloid-Derived Suppressor Cells

MDSCs were first identified by tumor biologists studying how the tumor microenvironment facilitates tumor evasion from the hosts anti-tumor immune response (38–40). These cells, which were initially defined as CD11b^+^Gr-1^+^ in mice, displayed robust immunosuppressive capabilities against the tumor-specific T cell response, creating an environment that allowed the tumors to grow unopposed (38–41). MDSCs have since been identified in a number of inflammatory settings in both human and mouse models, including infection, sepsis, trauma, auto-immunity, and transplant rejection (42–47). Given their immunosuppressive function, MDSCs have garnered particular interest in the field of transplant immunology as potential therapeutic tools to prevent graft rejection.

MDSCs can be subclassified into two main categories: monocytic MDSCs (M-MDSCs) and polymorphonuclear MDSCs (PMN-MDSCs, also referred to as granulocytic MDSCs), named for their phenotypic and morphologic similarities to monocytes and polymorphonuclear cells, respectively (48). The relative number of M-MDSCs and PMN-MDSCs has been shown to vary depending on cancer type and inflammation setting and can potentially be used to predict risk of graft versus host disease (49, 50). The importance of the ratio between M-MDSCs and PMN-MDSCs in the setting of organ transplantation has yet to be fully elucidated; however, limited data suggests that it is the monocytic subtype that predominates in mediating transplant tolerance (46, 51). Scalea et al. review some of the generally accepted surface markers of MDSCs, which vary between humans and mice (52). Human M-MDSCs can be characterized by dual expression of CD11b and CD14, as well as HLA-DR^low/−^ and lack of CD15 (52). These cells can be distinguished from mature human monocytes which share CD11b and CD14 expression but are HLA-DR− (48, 52). Human PMN-MDSCs, on the other hand, can be characterized by expression of CD11b and CD15 with no CD14 expression (48, 52). In humans, these cells have traditionally been distinguished from non-MDSC PMNs by density gradient centrifugation, but more recent studies have shown that LOX-1 expression may serve as a reliable marker to separate MDSC PMNs from non-MDSC PMNs *via* flow cytometry (48, 53). It has also been suggested that cytosolic calcium binding protein S100A9 expression can be used to further distinguish M-MDSCs from PMN-MDSCs *via* flow cytometry (54).

In mice, MDSCs are classically characterized by dual expression of CD11b and Gr1 (the myeloid lineage marker composed of Ly6C and Ly6G) (52, 55). Like human MDSCs, mouse MDSCs can be sub-classified as either M-MDSCs and PMN-MDSCs based on relative expression of Ly6C versus Ly6G (52, 55). M-MDSCs are characterized by high expression of Ly6C and lack of Ly6G (CD11b^+^Ly6G^-^Ly6C^high^), while PMN-MDSCs are characterized by expression of Ly6G and low levels of Ly6C (CD11b^+^Ly6G^+^Ly6C^lo^) (52, 55). Mouse M-MDSCs can further be distinguished from PMN-MDSCs based on the expression of CD49d on M-MDSCs (52).

MDSCs can be induced from hematopoietic stem cells under a variety of inflammatory conditions, as mentioned above. Normally, hematopoietic stem cells differentiate into common myeloid precursor cells (CMPs), which then further differentiate into immature myeloid cells (IMCs). In the absence of pathological inflammatory conditions, IMCs can migrate to secondary lymphoid organs and differentiate into mature macrophages, dendritic cells, or neutrophils (56). However, under the influence of mediators of chronic inflammation, these IMCs can develop into immunosuppressive MDSCs, which correlates with downregulation of interferon regulatory factor-8 (IRF-8) *via* a STAT3 transcription factor-dependent mechanism (56, 57). The main driver of MDSC expansion is G-CSF/GM-CSF, along with other pro-inflammatory mediators such as IL-2, IL-6, TGF-β, LPS, TNFα, IFN-gamma, and CXCL-1/2 (41, 48, 52, 58–60). In a study conducted by Marigo et al., the authors report that G-CSF, GM-CSF, and IL-6 could be used to rapidly generate functional MDSCs from human bone marrow precursor cells (58). However, they found that different combinations of these cytokines resulted in MDSCs with varying levels of tolerogenic activity, with MDSCs induced by a combination of GM-CSF^+^IL-6 possessing the highest tolerogenic activity (58). Interestingly, it has been shown that after MDSCs differentiate from precursor cells in the bone marrow, they can be maintained by activated T cells (61). IL-10 secreted from activated T cells promotes STAT3 phosphorylation on MDSCs, which subsequently leads to B7-H1 expression, a key molecule mediating MDSCs development and suppressor function (61). The phenotypic markers that delineate M-MDSCs and PMN-MDSCs in both mice and humans are summarized in **Table 1**.

### Regulatory B Cells (B10)

B cells classically play a central role in the adaptive immune response, most significantly as a component of humoral immunity; however, initial evidence that there exists a subset of B cells capable of down-regulating T cell-mediated inflammatory response came from studies with experimental autoimmune encephalomyelitis (EAE) in mice, showing that recovery from the Th1-driven autoimmune condition was dependent on B cells capable of producing IL-10 (62). In these studies, mice with selective IL-10 deficiency in the B cell compartment (but not the T cell compartment) exhibited a persistent type 1 autoimmune condition (62). In a similar murine model, lack of B cells resulted in delayed induction of Tregs in the CNS (63). Further investigation to elucidate the role of IL-10-producing B cells, termed “B10” cells, has shown that a phenotypically distinct CD1d^hi^CD5^+^CD19^hi^ B cell subset exists as a rare population of cells (1%–2% of all splenic B cells and 7%–8% of peritoneal B cells) that can be significantly expanded in the setting of T cell-mediated inflammation (64). Normally, B10 cells predominantly localize to the spleen and peritoneal cavity and are absent from the lymph nodes and peripheral blood (64, 65). Using a contact hypersensitivity (CHS) model in mice, Yanaba et al. showed that B10 cells exit the spleen and enter circulation and upregulate their IL-10 expression during the CHS response to downregulate the T cell response (64).

B10 cell development and maturation requires antigen receptor diversity, as transgenic mice with a fixed B cell receptor (BCR) exhibit 90% reduction levels of B10 cells (66). Further, both innate and adaptive signals can promote the expansion and maturation of B10 cells from B10 progenitor cells, most significantly by LPS and CD40L, respectively (65, 66). B10 development and activation appears to be T cell and pathogen-independent (65, 66). Of note, other regulatory B cells have been identified, including CD5^+^ B-1a cells, CD1d^+^ marginal zone B cells, and transitional-2-marginal zone precursor B cells (65). However, the bulk of regulatory B cell research focusses on the IL-10-competent CD1d^hi^CD5^+^CD19^hi^ subset (B10s) because these are responsible for the majority of B cell-derived IL-10 secretion and appear to be the most potent regulators of the T cell-mediated immune response in mice (65). Interestingly, TIM-1 (also known as Hepatitis A virus cellular receptor 1), a co-stimulatory molecule that regulates the immune response, has been identified as unique identifier of IL-10 producing regulatory B cells in mice (67). In a model of islet cell allograft transplant, TIM-1^+^ B cells were found to be highly enriched for IL-10 and IL-4 expression, and the subset of B cells expressing TIM-1 was significantly expanded (from 5%–8% up to 10%–15%) after allograft transplantation (67). These findings suggest that TIM-1 could be used as a unique marker to identify IL-10 competent regulatory B cells within other established subsets, such as the CD1d^hi^CD5^+^CD19^hi^ subset. In humans, cell surface markers CD24 and CD27 have been identified as additional identifiers of the B10 population (68). The phenotypic markers that delineate B10 cells in both mice and humans are summarized in **Table 1**.

**Table 1:** Phenotypic characterization of regulatory cells in mice and humans.

Cell	Mouse	Human
*Tregs*	CD4^+^ CD25^+^ Foxp3^+^	CD4^+^ CD25^+^ Foxp3^+^
*Tr1*	CD4^+^ CD49b^+^ LAG-3^+^ IL-10^+^ CD25^-^ Foxp3^-^	CD4^+^ CD49b^+^ LAG-3^+^ IL-10^+^ CD25^-^ Foxp3^-^
*M-MDSCs*	CD11b^+^ Gr-1^+^ Ly6G^-^ Ly6C^hi^ CD49d^+^	CD11b^+^ CD14^+^ HLA-DR^lo/−^ CD15^-^ S100A9^hi^
*PMN-MDSCs*	CD11b^+^Gr-1^+^ Ly6G^+^Ly6C^lo^ CD49d^-^	CD11b^+^ CD14^-^ HLA-DR^lo/−^ CD15^+^ LOX-1^+^
*B10*	CD1d^hi^ CD5^+^ CD19^hi^ TIM-1^+^	CD1d^hi^ CD5^+^ CD19^hi^ CD24^hi^ CD27^+^

## How They Exert Their Tolerogenic Effects

### Tregs

**Figure 1:**
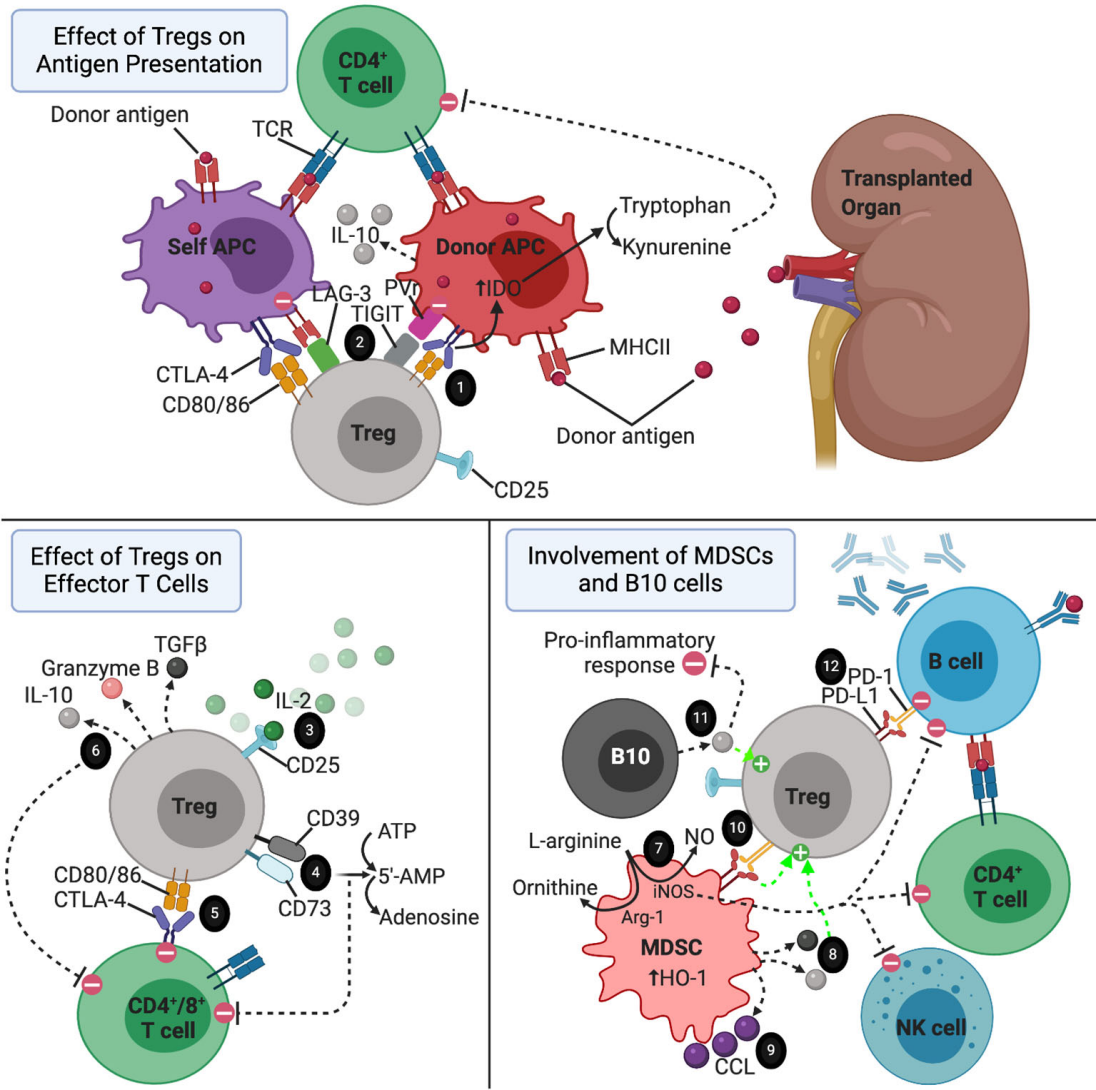
The immune environment surrounding a transplanted organ. 1) CTLA-4-CD80/86 interaction between Tregs and APCs resulting in increased tryptophan metabolism by APCs *via* IDO-dependent pathway. 2) LAG-3 and TIGIT on Tregs directing APCs towards a more tolerogenic phenotype. 3) Treg consumption of IL-2. 4) CD39 and CD73 acting as ectonucleotidases to break down ATP and 5’AMP to adenosine. 5) Tregs suppressing effector T cells *via* CTLA-4-CD80/86 interaction. 6) Tregs secreting anti-inflammatory cytokines to reduce the pro-inflammatory response, induce apoptosis of effector T cells, and promote the expansion of regulatory cell types. 7) MDSCs suppressing effector T cell, B cell, and NK cell proliferation *via* consumption of L-arginine in an iNOS dependent pathway. This mechanism is enhanced but upregulation of Arg-1 and HO-1 by MDSCs. 8) IL-10 and TGF-b secreted by MDSCs promoting the activation of Tregs. 9) CCL5 secreted by MDSCs establishing a graft-to-periphery gradient to recruit Tregs. 10) MDSCs promoting the suppressive function of Tregs *via* interaction between PD-L1 and PD-1. 11) IL-10 secreted by B10 cells promoting expansion of Tregs and exerting a broad array of anti-inflammatory effects. 12) Tregs inducing apoptosis of autoreactive B cells *via* interaction of PD-1 expressed on Tregs with PD-L1 on B cells.

Tregs have the ability to suppress the differentiation of naïve T cells into mature effector T cells, as well as suppress the functions of differentiated effector T cells and other players of the both the innate and adaptive immune systems, including B cells, macrophages, NK cells, and dendritic cells (21, 69). These tolerogenic effects are mediated through both cell surface molecules present on Tregs and soluble factors secreted by Tregs (**Figure 1**). One of the cell surface molecules that appears to play a central role in their immunosuppressive capabilities is CD25, a subunit of the IL-2 receptor (IL-2R), which is an important component of Treg differentiation and survival, as mentioned above. In addition to maintaining Treg homeostasis, the high levels and high affinity of IL-2R expression on Tregs results in IL-2 deprivation-mediated apoptosis of effector T cells, as IL-2 is also critical for the maintenance and survival of CD4^+^ and CD8^+^ effector T cells *in vitro* (69–72). However, *in vivo* studies have shown that IL-2 is actually not required for the maintenance of effector T cells, and that Tregs are able to exert their immunosuppressive effects even in mice that lack IL-2R on effector T cells (69). Another important contact-dependent mechanism by which Tregs suppress effector T cells *via* surface molecules involves an interaction between CTLA-4 on Tregs and CD80/86 on effector T cells (73–75). In addition to the direct interaction between Tregs and effector T cells, CTLA-4 on Tregs also interacts with CD80/86 present on the surface of dendritic antigen presenting cells (APCs) (76, 77). In this mechanism, engagement of CD80/86 ligands by CTLA-4, tolerogenic dendritic cells upregulate tryptophan metabolism *via* an indoleamine 2,3-dioxygenase (IDO)-dependent pathway, thereby inhibiting T cell proliferation (69, 76–78). Similarly, Tregs express PD-1, which has been shown to play an important role in suppressing autoreactive B cells in mice *via* interaction with PD-L1 expressed on B cells (79). CD39 and CD73 are two additional surface molecules on Tregs that suppress effector T cells by acting as ectonucleotides to convert ATP and 5′-adenosine monophosphate (5’-AMP), generated by pro-inflammatory cells like neutrophils, into adenosine, an anti-inflammatory molecule (80–82). Both human and mouse Tregs can also be induced to express high levels of the surface molecules, lymphocyte activation gene-3 (LAG-3) and TIGIT, which both exert their immunosuppressive effects primarily by interacting with APCs (83, 84). LAG-3 binds MHC II on DCs to suppress their antigen presenting capabilities, while TIGIT binds to poliovirus receptor on DCs to modulate their differentiation towards a more tolerogenic phenotype with enhanced IL-10 production (83, 84).

In addition to the surface molecules mentioned above, Tregs also secrete several soluble factors to exert contact-independent immunosuppressive functions. In a cytolytic mechanism of immunosuppression, Tregs secrete granzyme B to induce apoptosis of effector T cells and APCs (72, 85–87). This mechanism has been shown to be of particular importance in the maintenance of transplant tolerance (85). Tregs also secrete TGF-β and IL-10. As mentioned earlier, Tr1 cells are a subset of inducible Tregs that appear to be the main contributors of Treg-derived IL-10 production (36, 88, 89). The secreted IL-10 exerts broad immunosuppressive activity by downregulating MHC II and costimulatory molecules, suppressing the immunostimulatory capacity of APCs, and inhibiting the production of various pro-inflammatory cytokines by macrophages and DCs, overall resulting in reduced proliferation and activity of effector T cells (90–92). TGF-β secreted by Tregs appears to predominantly affect the cytolytic function CD8^+^ T cells while sparing CD4^+^ effector T cells (93–95). Given the importance of TGF-β signaling in the induction and activation of regulatory cell types, including Tregs and MDSCs, the TGF-β secreted by Tregs may also promote tolerance by enhancing these regulatory cell populations (23, 25, 32, 52).

### MDSCs

MDSCs, like Tregs, exert their immunosuppressive effects *via* a variety of both contact-mediated and soluble factor-mediated mechanisms (**Figure 1**). The primary targets of these mechanisms are effector T cells and NK cells (45, 96). One of the main mechanisms by which MDSCs act, especially in the setting of transplant tolerance, involves production of NO by inducible nitric oxide synthase (iNOS) (44, 45, 51, 97–100). This iNOS-dependent mechanism has a profound regulatory impact on effector T cells, B cells, and NK cells by suppressing the differentiation, proliferation, and various functions of these effector cell types (101). iNOS also suppresses T cell proliferation by consumption of L-arginine, an important substrate for T cell proliferation and the precursor substrate used by iNOS to produce NO (102). This mechanism is enhanced by arginase-1 (Arg-1), another enzyme that is upregulated by MDSCs which cleaves L-arginine to form ornithine and urea (97). MDSCs also upregulate hemoxigenase-1 (HO-1), and in a skin allograft transplant model using mice, MDSC-mediated T cell suppression and prolongation of graft survival was dependent on HO-1 expression (103).

MDSCs also have substantial interactions with Tregs, enhancing their migration, proliferation, and function (14, 41, 46, 51, 104–108). One of the main mechanisms involves an interaction between B7-H1 (PD-L1) on MDSCs and PD-1 expressed on Tregs (51, 108). In a murine model of islet cell transplantation, B7-H1 knockout mice were unable to exert their immunosuppressive capabilities or induce Tregs (108). Additionally, the presence of IFN-γ stimulates MDSCs to secrete IL-10 and TGF-β, thereby activating Tregs (41, 109). MDSCs also appear to play an interesting role in the setting of organ transplantation by establishing a graft-to-periphery gradient of CCL5 chemokine, which directs migration of Tregs from secondary lymphoid organs to the site of the graft in rat models of heart and kidney transplantation (106). Given these findings that MDSCs and Tregs act synergistically, it is reasonable to suggest that adoptive transfer of both MDSCs and Tregs together may provide a greater beneficial effect for achieving transplant tolerance than either one alone. To support this, in a model using MHC class II disparate allogeneic donor skin transplantation, mice receiving administrations of either G-CSF to induce MDSCs or IL-2 to induce Tregs resulted in prolonged survival of the graft, and the combination of both treatments resulted in even better survival of the graft (14). Interestingly, this same study showed that the induced MDSCs were more potent at suppressing T cell responses compared to naive MDSCs (14).

### B10 Cells

As their name implies, B10 cells predominantly exert their tolerogenic effects by producing and secreting the anti-inflammatory cytokine IL-10 in an antigen-specific manner (**Figure 1**). As mentioned above the in the setting of Tr1 cells, IL-10 suppresses the Th1 response, inhibits the antigen-present capabilities of APCs, and reduces the production and secretion of pro-inflammatory cytokines by macrophages and activated macrophages (65, 90, 91, 110). B10 cells have been shown to play a critical role in regulating the immune response in multiple models of autoimmunity in mice, including contact hypersensitivity, EAE, and collagen-induced arthritis (CIA) (62, 111, 112). In these models, adoptive transfer of CD40 mAb‐stimulated B cells reversed the autoimmune pathologies, while transfer of IL-10^-/-^ B cells had no effect, confirming the critical role of IL-10 production by B10 cells (62, 113).

Like MDSCs, B10 cells also promote tolerance by inducing the expansion of Tregs (67, 114, 115). One study demonstrated that human alloantigen-specific Foxp3-expressing Tregs can be generated in high frequencies by co-culturing CD4^+^CD25^-^ precursor T cells with CD40L-stimulated regulatory B cells (116). In a study investigating the role of B10 cells in the induction of oral tolerance, Sun et al. demonstrated that tolerance to a repeatedly administered antigen could be induced in mice in a Treg-dependent manner by transferring naïve T cells as long as IL-10-producing B cells were also present (117). Expansion of the antigen-specific Treg population, and therefore induction of tolerance, was absent in B cell-depleted mice, while co-transfer of B cells and naïve T cells into B cell-depleted mice restored the Treg population and resulted in tolerance (117). Like MDSCs mentioned above, these results suggest that co-transfer of both B10 and Tregs (or all three: B10, Treg, and MDSCs) in transplant patients could provide a synergistic therapeutic effect in the reduction of transplant rejection.

## Therapeutic Potential of Regulatory Cell Types in Transplant Models

### Tregs

Tregs have been extensively implicated as therapeutic options in a variety of organ transplant models, including skin, heart, kidney, islet cell, and lung (**Table 2**). The specific therapeutic strategy (*ex vivo* expansion versus *in vivo* induction, adjunctive immunosuppression, and specific subset of Tregs utilized) varies between studies, and it is likely that the optimal strategy may depend on the specific organ being transplanted. In a pivotal study published in Nature Medicine in 2010, Nadig et al. showed that *ex vivo* expanded CD127^lo^ Tregs could be adoptively transferred to inhibit the development of transplant arteriosclerosis (TA) in a clinically relevant chimeric humanized mouse system (12). This marked the first time that human Tregs were used to prevent TA in human arteries, which is the hallmark of chronic allograft dysfunction (12). In another recent study, Ratnasothy et al. demonstrated that exogenous administration of IL-2 lead to the preferential expansion of adoptively transferred donor-specific Tregs (specific for the MHC class I molecule K^d^), but not polyclonal Tregs, producing a synergistic effect that resulted in prolonged skin graft survival (from a mean of 13 days without treatment to 29 days with Tregs + IL-2) (118). In multiple other models of skin allograft in mice, Tregs were induced *in vivo* using exogenous administration of either interleukin-2 complex (IL-2C) or interleukin-33 (IL-33), resulting in prolonged survival of the skin grafts in the absence of immunosuppressive drug therapy (14, 118–120). In multiple models of kidney transplantation using non-human primates, adoptive transfer of *ex vivo* expanded donor-specific Tregs has been shown to prolong graft survival and prevent acute rejection (121, 122). Observational data has also suggested the potential efficacy of adoptive Treg therapy in human kidney transplant patients. In a retrospective study of human living donor kidney transplant recipients, flow cytometry analysis revealed significant increase in frequency of activated Tregs in the first 3 months after transplantation (123). Additionally, operationally tolerant kidney transplant patients have a higher frequency of more potent memory Tregs compared to patients with stable graft function or with chronic graft rejection, a trend which is also observed in operationally tolerant liver transplant recipients (124, 125).

**Table 2:** Animal transplant models utilizing regulatory cells.

Cell	Organ	Species	Cell Origin (recipient/donor/3^rd^ party)	Adjunctive Therapy	Mean Survival Time of Graft:Treatment vs. Control (Days)	Reference(Examples)
*Tregs*	Skin	Mouse; humanized mouse	Donor; recipient	IL-2 (118); IL-33 (119)	40 vs. 12 (14); 29 vs. 13 (118); >30 vs. 12 (119); 76 vs. 10 (120)	(14, 118–120)
	Heart	Mouse	Donor; recipient	IL-33 (15)	29 vs. 9 (15); >100 vs. 7 (16); 91 vs. 67 (126); >150 vs. 59 (146)	(15, 16, 126)
	Kidney	Nonhuman primate	Donor	Sirolimus (122)	416 vs. 22 (121); 48.5 vs. 22 (122)	(121, 122)
	Islet cell	Mouse; humanized mouse	3^rd^ party; recipient	Rapamycin + anti-CD8 (146)	32 vs. 17 (17); >60 vs. 15 (108)	(17, 108, 146)
	Lung	Humanized mouse	3^rd^ party;	N/A	Intimal thickening: 0.4% vs. 39.9% (127)	(127)
*Tr1*	Islet cell	Mouse	3^rd^ party	N/A	>100 vs. 25 (128)	(128)
*CAR-Tregs*	Skin	Mouse	3^rd^ party	N/A	>40 vs. 37 (129); 14 vs. 8 (131)	(129–131)
*MDSCs*	Cornea	Mouse	Recipient; 3^rd^ party	Glucocorticoids (135)	22.71 vs. 15.65 (43); 28.3 vs. 15.73 (136)	(43, 135, 136, 147)
	Skin	Mouse	Recipient; 3^rd^ party	G-CSF (14); IL-33 (119)	40 days vs. 16 days (14); 13.9 vs. 8.8 (43); 40 vs. 28 (59); >100 vs. 40 (99); >100 vs. 29 (109); >30 vs. 12 (119); 15 vs. 11 (132); 45 vs. 23.5 (133); 54.8 vs. 12.7 (148)	(14, 43, 59, 99, 103) (109, 119, 132, 133, 148)
	Heart	Mouse	Recipient; 3^rd^ party; donor	Rapamycin (105); anti-CD40L mAb (51); IL-33 (15)	29 vs. 9 (15); 67 vs. 7 (105); 58 vs. 10 (134)	(15, 51, 105, 134, 149)
	Islet cell	Mouse	Recipient; 3^rd^ party	N/A	>60 vs. 15 (100); >60 vs. 15 (104); >60 vs. 15 (108)	(58, 100, 104, 108)
*B10*	Islet cell	Mouse	3^rd^ party	Anti-TIM-1-mAb (67)	>100 vs. 15 (67)	(67)

Tregs have also been induced *in vivo* or adoptively transferred to prevent chronic rejection of heart transplants in mice (15, 16, 126). Takasato et al. demonstrated that donor-specific Tregs expanded *via* the indirect pathway were most effective in prolonging cardiac allograft survival (16). Interestingly, in the study conducted by Ma *et al*, low dose of the commonly used immunosuppressive drug sirolimus appeared to have a synergistic effect with Tregs promoting their expansion and homing to secondary lymphoid organs in the setting of heart transplantation (122).

In a humanized mouse model studying the role of Tregs in lung transplantation, adoptive transfer of allogenic human peripheral blood mononuclear cells enriched for Tregs resulted in significantly reduced transplant arteriosclerosis and intimal thickening (127). Finally, multiple studies have demonstrated the efficacy of using adoptively transferred human Tregs or inducing Tregs using adoptively transferred MDSCs to delay islet cell allograft rejection (17, 108). While minimal studies have utilized adoptive transfer of Tr1 cells in delaying graft rejection, the adoptive transfer of donor-specific (but not polyclonal) Tr1 cells has been shown to be efficacious in preventing islet cell allograft rejection (128).

Using chimeric antigen receptor (CAR) technology, multiple groups have developed Tregs expressing HLA-A2-specific CARs that have more potent immunosuppressive capabilities compared to polyclonal Tregs in the setting of humanized mouse models with HLA-A2^+^ skin xenografts, resulting in prevention of skin graft rejection (6, 129–131). Utilization of this technology overcomes several barriers associated with the use of natural Tregs. Namely, that the induction and expansion of antigen-specific Tregs involves a technically challenging protocol requiring repeated stimulation with the antigen of interest, which may not be feasible in the setting of clinical transplantation (129). These groups working with CAR technology have developed short transduction protocols that circumvent the need for extensive *in vitro* expansion (129). Additionally, these donor-specific CAR Tregs appear to be more specific and more potent than natural Tregs (129–131).

### MDSCs

The therapeutic role of adoptively transferred MDSCs has been extensively demonstrated in mouse models of skin transplantation (**Table 2**). Multiple groups have published protocols for inducing and activating MDSCs *in vitro* to be adoptively transferred into skin transplant recipients, including induction with LPS, TNF-α, human inhibitory receptor immunoglobulin-like transcript 2 (ILT-2), IFN-γ, or recombinant G-CSF, GM-CSF, or IL-6 (58, 59, 99, 103, 109, 132, 133). MDSCs induced *in vivo* with administration of G-CSF or IL-33 have also been shown to promote graft tolerance in skin transplanted mice (14, 119). Drujont et al. found that a single injection of LPS-activated MDSCs on the day of skin transplantation resulted in significant increase in survival of the graft, while repeated weekly injections resulted in even greater graft survival, suggesting that the full therapeutic potential of adoptive transfer of MDSCs may depend on repeated injections of activated MDSCs (59).

In heart allograft transplantation, both induced and adoptively transferred MDSCs have been successfully used to prolong graft survival in animal models. Garcia et al. demonstrated that donor MDSCs can be adoptively transferred and induced in the recipient by treatment with anti-CD40L mAb, resulting in MDSCs that migrate into the transplanted organ to prevent the initiation of the adoptive immune response and enhance the development of Tregs (51). Similarly to the skin transplant models described above, IL-33 has been used to induced *in vivo* expansion of MDSCs and Tregs to promote cardiac allograft survival in mice (15). Bryant et al. demonstrated that apoptotic donor splenocytes could be treated with the chemical cross-linker ethylcarbodiimide (ECDI) and preemptively infused into cardiac allograft recipient mice to induce MDSCs, resulting in long-term allograft survival (134).

He et al. found that sepsis-induced MDSCs could be harvested and adoptively transferred into mice immediately following corneal and combined corneal-skin transplantation, resulting in substantial expansion of MDSCs in the recipients bone marrow and in the corneal graft and increasing corneal graft survival from a mean of 15.65 days to 22.71 days (43). Glucocorticoids are known to induce expansion of MDSCs *in vitro*, and it has been shown that both systemic administration of glucocorticoids and adoptive transfer of glucocorticoid-induced MDSCs following corneal transplantation results in enhanced proliferation and mobilization of MDSCs, inducing immune tolerance (135). He et al. compared the tolerogenic capacities of inflammation-induced MDSCs versus tumor-induced MDSCs in the setting of corneal transplantation (136). In terms of reducing neovascularization and prolonging graft survival in the absence of immunosuppressive drugs, they found that inflammation-induced MDSCs were comparable to tumor-induced MDSCs when adoptively transferred to transplant recipients by retroorbital injection (136).

There is also extensive evidence to support the use of MDSCs to promote the survival of islet cell allografts. Marigo et al. demonstrated that MDSCs generated by treating bone marrow precursor cells with a combination of GM-CSF and IL-6 could be adoptively transferred to islet cell transplant recipients with four weekly injections immediately following transplantation (58). These MDSC’s inhibited the priming of CD8^+^ T cells and their adoptive transfer resulted in long term survival of allogenic islet cell transplant, with 75% of mice remaining euglycemic 200 days post-transplantation (58). MDSCs can also be generated *ex vivo* by co-culturing bone marrow precursor cells with GM-CSF, dendritic cells, and hepatic stellate cells (100). These MDSCs can be adoptively transferred to promote islet cell allograft survival in a manner that is dependent on iNOS expression and also results in the expansion and accumulation of antigen-specific Tregs in lymphoid organs close to the grafts when MDSCs are co-transplanted (100, 108).

In humans, MDSCs have been implicated as important regulators of tolerance in kidney and lung transplantation (46, 107, 137, 138). CD14^+^ M-MDSCs expand in renal transplant patients following transplantation, and these MDSCs are highly efficient in suppressing the proliferation of CD4^+^ T cells in mixed leukocyte reactions and are also capable of expanding Tregs *in vitro*. Additionally, there is a linear relationship between these MDSCs post-transplantation and circulating levels of Tregs (46, 138). In a study involving 50 patients with biopsy-proven acute T cell-mediated rejection (ATCMR), Meng et al. found that higher circulating levels of MDSCs post-transplantation correlated positively with allograft function and survival (107). *In vitro*, the MDSCs isolated from these patients were capable of expanding Tregs and inhibiting production of IL-17 (107). In a study investigating the role of MDSCS in human lung transplantation, it was found that circulating MDSCs are increased in stable lung transplant recipients versus non-transplant controls, and that patients with chronic lung allograft dysfunction (CLAD) had lower levels of MDSCs compared to stable recipients (137). These findings in humans, combined with the successful use of adoptive MDSC transfer in animal models described above, suggest that adoptive transfer of MDSCs could prolong organ allograft survival and promote graft tolerance in humans.

### B10

While studies involving the therapeutic use of B10 in transplantation are limited compared to Tregs and MDSCs, there is evidence implicating them in promoting tolerance in kidney, heart, skin, and islet cell transplantation (**Table 2**) (67, 139–144). In a mouse model of islet cell transplantation, anti-TIM-1 antibody was used to expand TIM-1^+^ B10 cells *in vivo* to significantly prolong islet cell allograft survival (67). Adoptively transferred TIM-1^+^ B10 cells exhibited potent tolerogenic activity in an antigen-specific fashion to prolong islet cell allograft survival while also enhancing the frequency of Tregs in the recipient (67). In multiple human and animal models of transplantation, including kidney, heart, skin, and islet cell, depletion of B cells during the period shortly following the transplant procedure when tolerance is being induced results in an enhanced T-cell response and accelerates graft rejection (140–143).

In human kidney transplant recipients, patients who achieve operational tolerance exhibit elevated levels of regulatory B cells compared to stable patients still requiring immunosuppression or patients with chronic rejection (139, 145). While there is a general paucity of studies directly investigating the adoptive transfer of B10 cells to promote tolerance, all of the above evidence suggests that B10 cells play an important role in inducing transplant tolerance and should be pursued as a potential therapeutic option alongside Tregs and MDSCs.

## Clinical Trials Involving Adoptive Transfer of Regulatory Cell Types

In recent years, a number of clinical trials have been initiated to study the use of adoptive cell therapy in organ transplantation. Thus far, these studies have focused on the use of Tregs, with a paucity of trials investigating MDSCs or B10 cells. Kidney and liver have been the main organs involved in these trials. A summary of all completed and ongoing clinical trails involving adoptive transfer of regulatory cell types in the setting of organ transplantation is presented in **Table 3**.

**Table 3:** Completed and ongoing clinical trials involving adoptive transfer of regulatory cell types.

Study	Phase	Condition	Intervention	Dosage	Status	Outcome
*NCT02145325*	1	Living donor renal transplant	Autologous polyclonal expanded Tregs	0.5, 1, 5 × 10^9^ cells	Complete	No adverse events related to therapy
*NCT02129881*	1/2	Living donor renal transplant	Autologous polyclonal expanded Tregs	1–10 × 10^6^ cells/kg	Complete	No adverse events related to therapy
*NCT02371434*	1/2	Living donor renal transplant	Autologous polyclonal expanded Tregs	0.5, 1, 2.5–3 × 10^6^ cells/kg	Complete	No adverse events related to therapy
*NCT02244801*	1	Living donor renal transplant	Autologous donor-alloantigen-specific Tregs	3, 9 × 10^6^ cells	Complete	No adverse events related to therapy
*NCT02091232*	1	Living donor renal transplant	Autologous donor-alloantigen-specific Tregs, cocultured with belatacept	N/A	Active	N/A
*NCT02088931*	1	Renal transplant	Autologous polyclonal expanded Tregs	320 × 10^6^	Complete	No adverse events related to therapy
*NCT02711826*	1/2	Renal transplant	Autologous polyclonal expanded Tregs	550 × 10^6^	Ongoing, recruiting	N/A
*ISRCTN-11038572*	2b	Living donor renal transplant	Autologous polyclonal expanded Tregs (TR001 cell product)	5–10 × 10^6^ cells/kg	Ongoing, recruiting (4/2020: recruiting suspended due to COVID-19)	N/A
*NCT03867617*	1/2	Living donor renal transplant	Autologous Tregs + donor bone marrow + Tocilizumab	N/A	Ongoing, recruiting (2019)	N/A
*NCT03943238*	1	Living donor renal transplant	Autologous expanded Tregs + donor HSC’s	25 × 10^6^ cells/kg	Ongoing, recruiting (2020)	N/A
*NCT01446484*	1/2	Living donor renal transplant in children	Autologous polyclonal expanded Tregs	2 × 10^8^ cells	Unknown (2011)	N/A
*NCT03284242*	1	Renal transplant in patients on Everolimus	Autologous polyclonal expanded Tregs	N/A	Ongoing, recruiting (2020)	N/A
*EUCTR2019-001730-34-NL*	1/2a	Living donor renal transplant	Autologous Antigen-Specific CAR-Tregs (TX200-TR101 cell product)	N/A	Ongoing, recruiting (2020)	N/A
*UMIN-000015789*	1/2	Living donor liver transplant	Autologous donor-alloantigen-specific Tregs	3.39 × 10^6^ cells/kg	Complete	No adverse events related to therapy. 7/10 patients achieved complete cessation of immunosuppression.
*NCT02166177*	1/2	Liver transplant	Autologous polyclonal expanded Tregs (TR002 cell product)	1, 4.5 × 10^6^ cells/kg	Complete	No adverse events related to therapy
*NCT02188719*	1	Liver transplant	Autologous donor-alloantigen-specific Tregs	50, 200, 800 ×10^6^ cells	Terminated	Difficulties in manufacturing the cell product
*NCT02474199*	1/2	Living donor liver transplant	Autologous donor-alloantigen-specific Tregs	400 × 10^6^ cells	Complete (2020)	N/A
*NCT01624077*	1	Living donor liver transplant	Autologous donor-alloantigen-specific Tregs	1 × 10^6^ cells/kg	Unknown	N/A
*NCT03577431*	1/2	Liver transplant	Autologous donor-alloantigen-specific Tregs with costimulatory blockade	2.5–500 × 10^6^ cells	Ongoing, recruiting (2019)	N/A
*NCT03654040*	1/2	Liver transplant	Autologous donor-alloantigen-specific Tregs	100–500 ×10^6^ cells	Withdrawn	Investigational product manufacturing challenges
*NCT03444064*	1	Islet cell transplant	Autologous expanded polyclonal Tregs	400–1600 × 10^6^ cells	Ongoing, recruiting (2020)	N/A
*NCT03162237*	N/A	Porcine islet cell xenotransplant	Autologous polyclonal Tregs	2 × 10^6^ cells/kg	Complete (2020)	N/A

### Kidney

A number of key phase I trials have been initiated investigating the use of regulatory cell types in kidney transplant recipients, including the TASK trial, the TRACT trial, and the ONE study (18, 150, 151). The TASK trial (NCT02711826) was conducted by researchers at UCSF to investigate the safety and feasibility of autologous polyclonal expanded Tregs in three patients with biopsy-proven subclinical graft inflammation at 6 months post-transplant (150). The group found no infusion reactions or serious adverse therapy-related events. The isolated Tregs received two rounds of stimulation with anti-CD3 and anti-CD28 beads and IL-2, along with deuterated glucose to label and track the cells (150). While the patients were maintained on an immunosuppressive regiment of tacrolimus, mycophenolate mofetil, and prednisone, the infused Tregs demonstrated persistence and stability comparable to non-immunosuppressed subjects infused with the same dose of Tregs (150). These results have set the stage for future trials testing the efficacy of polyclonal and antigen-specific Tregs in the setting of subclinical inflammation in renal transplants (150).

In the TRACT trial (NCT02145325), a group from Northwestern University performed a dose-escalation trial in living donor renal transplant recipients, with three dosing tiers (0.5, 1, and 5 × 10 (9) cells) and three recipients per dose (18). The infused Tregs exhibited high purity (>98% CD4^+^CD25^+^) with high stability of the Foxp3 promoter. *In vivo*, the infused Tregs resulted in sustained, elevated levels of circulating Tregs. Like the TASK trial, this trial reported no adverse events related to the therapy up to 2 years post-transplant when the results were published, providing the necessary safety data move the trial into phase II efficacy studies (18).

The ONE study involved seven single-arm trials conducted at eight different institutions in across five countries, investigating the use of cell-based protocols to reduce general immunosuppression in living-donor renal transplant recipients (151). The cell-based protocols utilized in the various trials included two polyclonal Treg products (NCT02371434, NCT02129881) and two donor-antigen reactive Treg products (NCT02244801, NCT02091232), as well as one tolerogenic dendritic cell and one regulatory macrophage cell product (151). The two polyclonal Treg products, pTreg-1 (NCT02371434) and pTreg-2 (NCT02129881), were isolated and expanded using protocols published by Fraser et al. and Landwehr-Kenzel *et al*, respectively (19, 152). One of the donor-specific Treg products utilized conditions of costimulatory blockade (NCT02091232) while the other product was generated by stimulating recipient PBMCs with donor B cells that had been activated by human CD40L expressed on K562 cells (NCT02244801) (20, 153). All Treg products were delivered as a single intravenous infusion within 10 days following the day of the transplant procedure, and all patients were routinely monitored for the primary endpoint of biopsy-confirmed acute rejection (BCAR) within 60 weeks following transplantation. Combined data across all of the cell-based therapy groups revealed no safety concerns compared to the standard immunosuppressive treatment group, and the cell-based groups experienced lower infection rates. Additionally, rates of BCAR were comparable between the standard immunosuppressive group and the cell-based therapy group (12% vs. 16%), overall suggesting that adoptive transfer of Tregs could be a useful therapeutic tool for preventing rejection in renal transplant patients while reducing the burden of immunosuppression.

The STEADFAST study (EUCTR2019-001730-34-NL), a recently initiated phase I/IIa trial, has been initiated in the U.K. and the Netherlands to evaluate the safety and tolerability of an autologous HLA-A2-specific Treg therapy (TX200-TR101 product) in living donor renal transplant recipients. This will be the first clinical trial investigating the use of a CAR-Treg therapy in the prevention of transplant rejection in humans. As such, the results of this study are highly anticipated.

### Liver

In 2016, Todo et al. published a pilot study on the use of adoptive transfer of donor-specific Tregs in 10 living donor liver transplant patients (UMIN‐000015789) (154). Donor alloantigen-specific Tregs were generated *in vitro* by coculturing recipient lymphocytes with irradiated donor cells along with anti-CD80/86 mAbs for 2 weeks. These Tregs demonstrated donor-specific inhibition in a mixed lymphocyte reaction and were infused in all 10 patients without any significant adverse events. After transplantation and infusion with Tregs, patients underwent splenectomy and were subsequently weaned off of traditional immunosuppression of mycophenolate mofetil and tacrolimus starting at 6 months until complete cessation at 18 months. The ultimate goal of stable graft function with complete discontinuation of immunosuppression after 18 months was achieved in seven out of the 10 patients, while the other three patients developed mild rejection during the weaning period and were continued on low dose immunosuppression. Of note, these three patients all had autoimmune liver disease.

Several ongoing studies are also utilizing donor alloantigen-specific Tregs in the setting of liver transplantation, including the LITTMUS trial (NCT03577431 and NCT03654040), the ARTEMIS trial (NCT02474199), and the deLTa trial (NCT02188719). The first arm of the LITMUS trial (NCT03577431) involved the use of donor alloantigen-specific Tregs cultured and stimulated in the presence of costimulatory blockade, while the second arm of the study (NCT03654040) intended to use Tregs without costimulatory blockade but was withdrawn due to difficulty manufacturing the cell product. The deLTa trial set out to give three cohorts three different doses of donor alloantigen-specific Tregs (50, 200, and 800 ×10 (6) cells) but the study was terminated due to difficulties manufacturing the cell product. The ARTEMIS trial, which specifically investigated the use of Tregs in weaning patients off of calcineurin inhibitors (CNIs), was completed in January 2020 and results are still pending.

Trials involving liver transplant recipients have also investigated the use of polyclonal Tregs. One of these studies, known as the ThRIL trial (NCT02166177), was a phase I/IIa trial evaluating the safety, tolerability, and efficacy of polyclonal expanded Tregs and was completed in January 2018 (155). This study utilized a CliniMACS-based cell isolation protocol and expanded the Tregs using a co-culture containing anti-CD3/CD28 beads, IL-2, and rapamycin (155). Preliminary safety data from this trial was presented in abstract form at the 2017 American Transplant Congress meeting, reporting no dose-limiting toxicities in patients receiving the polyclonal Tregs (156). Results are still pending regarding the efficacy of the treatment.

### Islet Cell

Two studies are currently being conducted to investigate the use of polyclonal Tregs to induce tolerance of islet cell allografts and xenografts (NCT03444064, NCT03162237) in patients with type 1 diabetes. Results of both of these studies are still pending.

## Discussion

The bulk of research published so far on adoptive cell therapy in the setting of solid organ transplantation has implicated regulatory cell types as potential therapeutic options for reducing the burden of systemic, lifelong immunosuppression in transplant recipients. As described in this review, each of these cell types have distinct mechanisms by which they exert their tolerogenic effects; however, there is also considerable interaction between these cell types. To date, the vast majority of animal model studies and all of the clinical trials have utilized the adoptive transfer of only a single cell type. Given the synergistic effect that these cells exert on one another, we suggest further investigation into using the adoptive transfer of multiple cell types together to induce tolerance in a single transplant recipient. This will require further research into the optimal combinations, ratios, and timing of when to transfer these cells. Additionally, thus far, clinical trials utilizing the adoptive transfer of regulatory cell types in the setting of solid organ transplantation have focused almost exclusively on Tregs. We suggest that further studies be conducted using other regulatory cell types, such as MDSCs and B10 cells, given the promising data that has been generated with their use in animal models.

Other questions that require answering before adoptive cell therapy can become a widely utilized therapeutic approach in transplantation include the optimal timing, dosing range, and dosing frequency for the different cell types and for the different organs being transplanted. This will require large-scale studies with multiple cohorts to be able to accurately compare the different strategies. Similarly, further research should be conducted to establish the most efficient and universally applicable isolation and expansion protocol for each of the different cell types, especially given that the requirement of repeated, prolonged antigen stimulation to produce antigen-specific Tregs is a limitation in many settings. This may require further pursuit of the use of CAR technology to design CAR-Tregs for clinical trials.

Immunoengineering offers promising new avenues for optimizing adoptive cell therapy. Using gene transfer technology, Tregs can be transduced with antigen-specific TCRs or CARs. While already widely used in the treatment of hematological malignancies, CAR-T cells have gone through multiple generations of optimization to increase their efficacy and limit off-target toxicities. Similar optimizations will be required for CAR-transduced Tregs before they can be widely applied to human transplant patients, including optimal co-stimulatory molecules, appropriate antigen specificity (including CARs with bi-specificity), and the possible inclusion of suicide genes to improve the safety profiles of these therapies. The high cost of these engineered cell products is also a barrier that will need to be addressed moving forward.

It should be noted that other regulatory cell types exist and have shown promise as potential therapeutic tools, including tolerogenic dendritic cells, natural killer cells, and regulatory macrophages. As the novel field of adoptive cell therapy continues to grow, these cells may emerge as important players along with Tregs, MDSCs, and B10 cells. Overall, the therapeutic potential of regulatory immune cells in the setting of solid organ transplantation is incredibly promising and will be exciting to follow as the foundational research outlined in this review is translated to the clinic.

## Author Contributions

All authors contributed to the article and approved the submitted version.

## Funding

The authors were supported by NIH/NIAID grant 1R01AI142079-01A1 (SN) and NIH/NHLBI 1RO1 HL140470-0181 (CA), as well as the Patterson-Barclay Memorial Foundation.

## Conflict of Interest

The authors declare that the research was conducted in the absence of any commercial or financial relationships that could be construed as a potential conflict of interest.

## Abbreviations

AMR, Antibody-mediated rejection; APC(s), Antigen presenting cell(s); B10 Cell, IL-10 secreting B cell; BCR, B cell receptor; CAR, Chimeric antigen receptor; EAE, Experimental autoimmune encephalomyelitis; iNOS, Inducible nitric oxide synthase; iTreg, Induced Treg; M-MDSC(s), Monocytic MDSC(s); MDSC(s), Myeloid derived suppressor cell(s); NK Cell, Natural killer cell; PMN-MDSC(s), Polymorphonuclear (granulocytic) MDSC(s); TCR, T cell receptor; Tr1 Cell, T regulatory type 1 cell; Treg(s), Regulatory T cell(s).
